# The species, density, and intra-plant distribution of mites on red raspberry (*Rubus idaeus* L.)

**DOI:** 10.1007/s10493-024-00930-7

**Published:** 2024-06-27

**Authors:** Jiunn Luh Tan, Nina Trandem, Zhibo Hamborg, Bijaya Sapkota, Dag-Ragnar Blystad, Jana Fránová, Rostislav Zemek

**Affiliations:** 1 Department of Zoology, Faculty of Science, University of South Bohemia, České Budějovice, 37005 Czech Republic; 2 Institute of Entomology, Biology Centre CAS, České Budějovice, 37005 Czech Republic; 3 Division of Biotechnology and Plant Health, Norwegian Institute of Bioeconomy Research (NIBIO), 1433 Ås, Norway; 4 Institute of Plant Molecular Biology, Biology Centre CAS, České Budějovice, 37005 Czech Republic

**Keywords:** Anystidae, *Emaravirus idaeobati*, Eriophyidae, Generalist predatory mites, Raspberry leaf blotch virus, Tetranychidae

## Abstract

The adoption of the European Green Deal will limit acaricide use in high value crops like raspberry, to be replaced by biological control and other alternative strategies. More basic knowledge on mites in such crops is then necessary, like species, density, and their role as vectors of plant diseases. This study had four aims, focusing on raspberry leaves at northern altitude: (1) identify mite species; (2) study mite population densities; (3) investigate mite intra-plant distribution; (4) investigate co-occurrence of phytophagous mites, raspberry leaf blotch disorder and raspberry leaf blotch virus (RLBV). Four sites in south-eastern Norway were sampled five times. Floricanes from different parts of the sites were collected, taking one leaf from each of the upper, middle, and bottom zones of the cane. Mites were extracted with a washing technique and processed for species identification and RLBV detection. Mites and leaves were tested for RLBV by reverse transcription polymerase chain reaction (RT-PCR) with virus-specific primers. Phytophagous mites, *Phyllocoptes gracilis*, *Tetranychus urticae*, and *Neotetranychus rubi*, and predatory mites, *Anystis baccarum* and *Typhlodromus* (*Typhlodromus*) *pyri* were identified. All phytophagous mites in cultivated raspberry preferred the upper zone of floricanes, while in non-cultivated raspberry, they preferred the middle zone. The presence of phytophagous mites did not lead to raspberry leaf blotch disorder during this study. RLBV was detected in 1.3% of the sampled plants, none of them with leaf blotch symptoms, and in 4.3% of *P. gracilis* samples, and in some spider mite samples, implying that Tetranychids could also be vectors of RLBV.

**Supplementary Information:**

The online version contains supplementary material available at 10.1007/s10493-024-00930-7.

## Introduction

Mites (Acari) can be predators, herbivores, detritivores, fungivores and parasites (Dhooria 2016; O’Connor 2009). There are about 7000 known species of phytophagous mites, of which approximately half are in the superfamily Eriophyoidae and the other half in the superfamily Tetranychoidea and family Acaridae (Dhooria 2016). Many of them are known as economically important pests in agricultural crops, causing not only physical damage but also acting as vectors of plant pathogens like viruses. Acaricides have an important role in managing the population of these mites worldwide, and most of these acaricides are used in fruit and vegetable productions (Leeuwen et al. 2015). However, the adverse effects of pesticides on the environment and human health have led to increasingly stricter regulations to reduce pesticide dependence (Buckwell et al. 2020). The European Union aim to reduce the use of pesticides by 50% within 2030 through the adoption of Farm to Fork Strategy as part of the European (EU) Green Deal. (European Commission 2020). This will further limit the number of acaricides in minor crops, such as raspberry, and making it even more vital to succeed with biological control and other alternative strategies. Several factors need to be considered and better understood to implement biological control efficiently, among them species and density of both phytophagous and predatory mites naturally occurring on the plants. In addition, knowledge on the most common sites of infestation of these phytophagous mites on the plants is crucial to improve the precision of management approaches, such as application of biopesticides and release of commercial natural enemies.

To date, at least 18 species of phytophagous mites from the families of Tetranychidae, Tenuipalpidae and Eriophyidae have been reported on raspberry (Tan et al. 2022). However, the mite species composition on raspberry will differ with geographical region since most species are not distributed across all continents or climates. For instance, *Tetranychus urticae* Koch (Acari: Tetranychidae) is a cosmopolitan pest but *Amphitetranychus viennensis* (Zacher) (Acari: Tetranychidae) is only found in two continents, Asia and Europe (CABI 2021; Migeon and Dorkeld 2023). This shows the importance to study the mite species composition in different geographical regions to properly manage them. The feeding of phytophagous mites causes physical damage on raspberry, and the symptoms of such damage vary between different families of mites. For instance, the presence of spider mites, family Tetranychidae, on raspberry usually can be noticed first as a light, stippled appearance on the adaxial leaf surface and the leaf will eventually turn yellow, silver or bronze. This is because they feed by sucking the sap, removing chlorophyll in the process. Heavy infestations on primocanes will cause early leaf fall and stunted growth of canes and leaves, affecting the crop potential; on floricanes, it will cause yield losses and lower fruit quality (Fisher 1991). On the other hand, the infestation of raspberry leaf and bud mite, *Phyllocoptes gracilis* (Nalepa) (Acari: Eriophyidae), may result in chlorotic spots and blotches which may eventually turn into reddish necrotic areas on leaves, hairless spots on the abaxial surface of leaves, shoot proliferation, misshapen fruits, uneven ripening and coloration of drupelets, stunted growth, curled and occasionally distorted leaves, and in some cases, small galls may be observed (Fisher 1991; Gordon and Taylor 1976; Milenković and Marčić 2012).

Among all the mites on raspberry, only *P. gracilis* is known to be the vector of a raspberry virus - raspberry leaf blotch virus (RLBV, species: *Emaravirus idaeobati*; genus: *Emaravirus*; family: *Fimoviridae*) (Dong et al. 2016; ICTV 2023; McGavin et al. 2012). Before the discovery of this virus, the yellow blotch symptoms were known as the raspberry leaf blotch disorder and thought to be solely caused by the feeding of *P. gracilis* (Gordon and Taylor 1976). But about a decade ago, McGavin et al. (2012) detected RLBV in raspberry leaves with both symptoms of yellow blotches and feeding *P. gracilis*. Thenceforth, raspberry leaf blotch disorder has been attributed to both *P. gracilis* and RLBV infections. However, to date, there is still no definite determination if the yellow blotch symptoms are caused by the feeding of mites or by RLBV infections.

In this study, four main objectives, focusing on raspberry leaves in south-eastern Norway, had been formulated: (1) to identify the species of phytophagous and predatory mites, (2) to study the density of these mites on cultivated raspberry as well as non-cultivated raspberry, (3) to investigate the intra-plant distribution of phytophagous mites, and (4) to investigate the co-occurrence of phytophagous mites, raspberry leaf blotch disorder and RLBV. This information could assist in the development of better mite management strategies in raspberry production.

## Materials and methods

### Study sites and leaf sampling

Raspberry (*Rubus idaeus*) leaves were sampled five times from June to early August 2022 in Viken county, Norway. There was a total of four sampling sites (Table 1), with the latitude ranging from 59°41’37.441” N to 59°20’17.577” N and the longitude from 10°46’30.532” E to 10°54’36.081” E. Two sites consisted of semi-natural boundary vegetation with non-cultivated raspberry and two sites were at commercial farms with cultivated raspberry of the cultivar ‘Glen Ample’, approximately eight years old. One farm used high polytunnels and the other was open field. Both farms also had non-cultivated raspberry present in the boundary vegetation. The non-cultivated raspberry may include wild raspberry as well as volunteers of unknown raspberry cultivars. In both raspberry farms, the grower applied some plant protection products to suppress pests and diseases in the cultivated raspberry. In the open field site, the synthetic pyrethroid, lambda-cyhalothrin (Karate® 5 CS, Syngenta), was applied once at recommended dosage before flowering and sampling (approximately 3^rd^ June 2022), while the fungicide chlorothalonil (Geoxe, Syngenta) was applied twice (25^th^ June and 7^th^ July) at recommended dosage during the sampling period. None of the pesticides applied was to target phytophagous mites. In contrast, no synthetic pesticides were used by the farmer at the site with tunnel cultivated raspberry. Instead, he had applied vegetable oil with soap during autumn of the previous year to target overwintering eriophyid mites. He also released the phytoseiid predatory mite, *Neoseiulus cucumeris* (Oudemans) (Acari: Phytoseiidae) (Amblyline, Bioline AgroSciences, United Kingdom) during late spring, as recommended by the advisory service to prevent mite damage.

All raspberry leaves sampled were composite leaves, consisting of three leaflets. The leaves were collected only from floricanes, sampling three leaves from each cane; one from the bottom third of the cane, one from the middle third, and one from the upper. Every leaf was put separately in plastic bags with a piece of damp tissue. Primocanes were not sampled because, in June, they were too small to sample for intra-plant distribution study.

In the two sites with only non-cultivated raspberry, each site was divided into equal sections, and at each sampling, one plant was randomly selected for leaf sampling in each section. The number of sections depended on the size of the sampling site. A total of four and three non-cultivated plants were sampled from the two sites respectively per sampling. In Utveien (Site 2, Table 1), only four samplings were carried out because the site was discovered after the first sampling.

In the two sites with cultivated raspberry, three rows were selected with an equal interval between them, to evenly cover the sampling area. In each row, three plants were randomly selected, one at each end of the row (about 1 m from the end) and one in the middle of the row. In addition, three non-cultivated raspberry floricanes from the boundary vegetation were selected at random and three leaves collected from each plant in the same manner. These non-cultivated plants were less than three meters away from the cultivated open-field raspberry, and 14 to 160 m away from the cultivated raspberry under tunnel. Thus, a total of nine cultivated and three non-cultivated floricanes were sampled from each of these two sites per sampling, except only two non-cultivated floricanes were sampled in Råde (Site 4, Table 1) in the first sampling due to limited number of leaves available.

All the leaf samples were stored in a 4 °C refrigerator for less than 8 h until they were processed. In total, 453 leaves were sampled from 151 plants.

**Table 1:** The sampling sites with types of raspberry, acreage, number of samplings and number of plant samples collected at each site

Site	Name of site	ToF^†^	Area (ha)	No. of samplings	Date of samplings	Total sample collected	
						Plants	Leaves
1	Hogstvetveien, Ås	NC	0.018	5	8, 21 Jun, 5, 19 Jul, 2 Aug	20	60
2	Utveien, Ås	NC	0.003	4	23 Jun, 7, 21 Jul, 3 Aug	12	36
3	Kråkstad	OF	1.286^‡^	5	13, 24 Jun, 11, 25 Jul, 5 Aug	45	135
		NC	0.008	5		15	45
4	Råde	TN	0.659	5	14, 24 Jun, 11, 25 Jul, 5 Aug	45	135
		NC	0.009	5		14	42

^†^ToF: Type of field, NC: non-cultivated raspberry (semi-natural boundary vegetation), OF: cultivated open-field raspberry, TN: raspberry cultivated under tunnel

^‡^This is the full area of the Kråkstad field, but only about half of the field (∼ 0.6 ha) was sampled

### Extraction and counting of mites

Before extraction of mites, the leaf area was estimated using a free mobile application LeafArea (version 2.1.6) by Skyberry available in Google Play store. The leaf was placed on a white paper accompanied by a plastic ruler. The photo of the leaf with the ruler was taken with a mobile phone (Huawei P30 Pro, China). This photo was uploaded to the LeafArea application, and the calibration was done by specifying the scale of 1 cm with the ruler in the photo. The area of the leaf was then estimated automatically by the application. All relevant symptoms on the leaves were also recorded by a written description of the symptoms, such as mosaic or yellowing, in addition to the photo of all leaves.

**Fig. 1:**
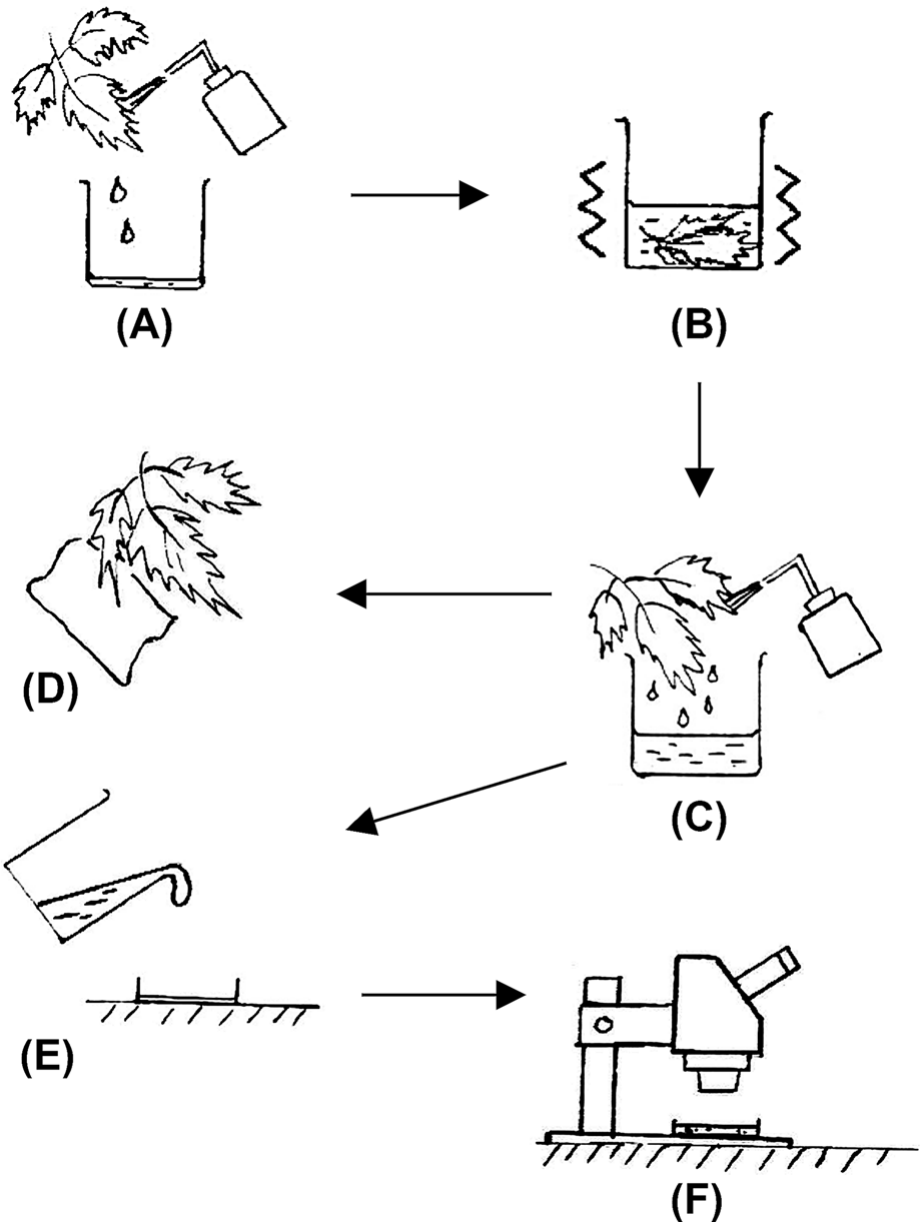
Extraction of mites from leaf samples. (**A**) flushing the leaf with 70% ethanol, (**B**) shaking with ethanol in beaker, (**C**) reflushing leaf with 70% ethanol, (**D**) drying the washed leaf with tissue paper, (**E**) transferring the ethanol to a Petri dish, (**F**) observation

The washing technique described in Pérez-Moreno and Moraza-Zorrilla (1998) was modified to extract mites from the leaf samples (Fig. 1). Each leaf was flushed individually with 70% ethanol (diluted from 96% ethanol, VWR chemicals, France) by using a wash bottle. The ethanol was then collected with a beaker, either 250 ml or 500 ml (depending on the size of the leaf), and the leaf was submerged in it. The ethanol was topped up if the volume was insufficient to cover the leaf, and the beaker shaken with a horizontal motion by hand for approximately 30 s. The leaf was removed from the beaker and flushed with 70% ethanol again using a wash bottle before drying the leaf with a tissue paper to avoid overexposure to ethanol. The leaf was stored in a refrigerator (4 °C, for less than 8 h) until further processing for virus detection. The ethanol in the beaker was transferred into a plastic Petri dish (size depending on the volume of ethanol) and all the mites were counted under a binocular stereomicroscope (Leica MZ7.5, Germany) at 32× to 40× magnification. The counts were performed by placing a black sheet with a millimeter grid beneath the dish.

After counting, the mites were collected using a 10–100 µl micropipette (Finnpipette®, Thermo Scientific, USA) into a dry Petri dish which was left open for the ethanol to evaporate. The mites were then collected using a minuten pin and either put into 70% ethanol to preserve for morphological identification or into 100 µl of DNA/RNA Shield™ buffer (Zymo Research, USA) for molecular identification and RLBV detection. The mites collected in DNA/RNA Shield™ buffer were stored at -20 °C if they were not extracted on the same day. The mites from leaves of the same plant were pooled for identification purposes. To succeed with molecular identifications, at least 30 eriophyid mites, 20 spider mites or five predatory mites per pooled sample were deemed necessary, except for bigger mites like Anystidae, for which one individual was sufficient. Samples with less mites were kept for morphological identification.

### Identification of mites

For molecular identification, the mites were crushed using a small glass rod, and further crushed after adding 500 µl of TRIzol® reagent (ZYMO Research, USA). Subsequently, the total RNA of the mites was extracted using Direct-zol™ RNA MiniPrep extraction kit (Zymo Research, USA). The concentration and purity of the extracted total RNA were measured using Nanodrop™ 2000 spectrophotometer (Thermo Scientific, USA) and stored at -20 °C for a short period during preparation for subsequent step, and then stored at  -80 °C for long term storage. The extracted RNA was converted to cDNA using Superscript IV Reverse Transcriptase (Thermo Fisher Scientific, USA) following the manufacturer's instructions. Subsequently, the cDNA was amplified through polymerase chain reaction (PCR) using cytochrome c oxidase subunit I (*COI*) primers as internal control and molecular species identification (Folmer et al. 1994; Ovalle et al. 2020) (Table 2). The PCR master mix was prepared by mixing 2.5 µl of 10X Buffer, 1 µl of 10 mM dNTP, one unit of Taq polymerase (Invitrogen™, ThermoFisher Scientific, USA), 17.8 µl of RNase/DNase-free water, 1 µl of each forward/reverse primer (10 mM concentration). After that, 2 µl of cDNA template was added. Positive control (cDNA template prepared from a plant previously confirmed to be infected with RLBV) and negative control with no template (MQ water) were included in all reactions. The PCR programs applied were as follows: initial pre-denaturation step at 95 °C for 2 min, followed by 35 cycles of denaturation at 95 °C for 30 s, annealing at 47 °C for 30 s, and extension at 72 °C for 45 s. A final extension was performed at 72 °C for 7 min. Each resulting PCR product (10 µL) was subjected to electrophoresis within a 1.5% agarose gel that had been pre-stained with SYBR safe DNA stain (Invitrogen™, ThermoFisher Scientific, USA). A 100 bp ladder was applied and pictures were analyzed using the Quantity One software (Bio-Rad Laboratories Inc., USA). Amplicons that yielded a product of appropriate size were sequenced using Sanger sequencing (Eurofins Genomics). All the sequences were analyzed using Molecular Evolutionary Genetics Analysis (MEGA) version 11 software (https://www.megasoftware.net/) (Tamura et al. 2021) and identified using the Basic Local Alignment Search Tool (BLAST) database provided by the National Center for Biotechnology Information (NCBI).

**Table 2:** Primer used for amplification of cytochrome c oxidase subunit I gene (*COI*) sequences

Name	Primer name	Sequence (5`-3`)	AT^†^	Ps^†^
COI	LCOI490	GGTCAACAAATCATAAAGATATTGG	47 °C	710
	HCO2198	TAAACTTCAGGGTGACCAAAAAATCA		

^†^The primers were in reference to Folmer et al. (1994); AT: Annealing temperature; Ps: Product size in base pair (bp)

For morphological identification, the mite samples were transferred from the 70% ethanol into 80% lactic acid (Lach-Ner, Czech Republic) for clearing. Eriophyid and predatory mites were placed in lactic acid on microscope slides and heated on a hot plate for 1–2 min at 80 °C to clear the mites before placing the cover glass, on which lactic acid was also the mounting medium. Lactic acid was used as mounting medium because it is good for temporary slides which suited our purpose. The mounted samples were then left undisturbed at room temperature for at least two days. Only adult females of eriophyid and predatory mites were used for identification. In the case of spider mites, these were cleared in the lactic acid at room temperature for at least 48 h before being mounted on microscopic slides ringed with paraffin, using Hoyer’s solution (Entomopraxis, Barcelona, Spain) as mounting medium. After positioning the mites on a paraffin ringed microscopic slide, the slide was heated briefly at 60–80 °C to melt the paraffin and fix the mite position. This procedure was carried out because adults of both sexes were necessary for identification, in which males have to be positioned on the lateral side to have a clear observation on the aedeagus. The paraffin ring was added to the slides to prevent crushing the spider mites by the cover glass. Hence, Hoyer’s solution was used as mounting medium because lactic acid does not separate well with the paraffin after heating. The mounted mites were examined and identified using the Carl Zeiss Jena Peraval Interphaco microscope (Germany) and Olympus BX51TF microscope (Japan). For spider mites, the keys of Maric et al. (2018), Ben-David et al. (2013), Gutierrez (1985), Trägardh (1915) and Pritchard and Baker (1955) were used, while for eriophyid mites, the key in Xue et al. (2009) and Pye and Lillo (2010) were used. For predatory mites, the keys of Cuthbertson et al. (2014), Meyer and Ueckermann (1987) and Miedema (1987) were used.

### Raspberry leaf blotch virus (RLBV) detection in plants and mites

The three leaf samples from each plant, washed as described above, were pooled and ground with liquid nitrogen to begin the RNA extraction process. The samples were pooled because RLBV can move systemically in red raspberry. Total RNA from leaf samples were extracted with the Plant/Fungi Total RNA Purification Kit (Norgen Biotek Corp., Canada). The concentration and purity of the extracted total RNA from leaf samples were measured using Nanodrop™ 2000 spectrophotometer (Thermo Scientific, USA) and stored at -20 °C for a short period during preparation for subsequent step, and then stored at -80 °C for long term storage. The cDNA synthesis and RLBV detection with PCR using the specific primer were performed as described in the ‘Identification of Mites’ section. As an internal control for amplification for leaf samples, RT-PCR was employed to amplify the mitochondrial NADH dehydrogenase nad5 mRNA (Menzel et al. 2002). To detect RLBV in mites, the same extracted RNA from the molecular identification of mites (refers to ‘Identification of Mites’ section) was used. Only samples that tested positive with *COI* primers were further proceed for RLBV detection with RLBV specific primers (Table 3).

**Table 3:** Primers used for amplification of internal control and detection of raspberry leaf blotch virus

Name	Primer name	Sequence (5`-3`)	AT^†^	Ps^†^
NAD	NAD-F	GATGCTTCTTGGGGCTTCTTGTT	50 °C	181
	NAD-R	CTC CAG TCA CCA ACA TTG GCA TAA		
RLBV	RLBV_F	ATCCAGTAGTGAACTCC	56 °C	560
	RLBV_R	CACCATCAGGAACTTGTAATGTTT		

^†^The primers were in reference to Menzel et al. (2002) for NAD and Dong et al. (2016) for RLBV; AT: Annealing temperature; Ps: Product size in base pair (bp)

### Statistical analysis

The general population density of mites were plotted in a graph, in unit of mites per square centimeter (cm^2^) of leaf area. The intra-plant distribution of eriophyid and spider mites were analyzed using Poisson generalized linear mixed models (GLMMs). The GLMMs were carried out using the statistical software R, version 4.2.1 (The R Foundation, https://www.r-project.org/). The package *lme4* was used for all mixed models (Bates et al. 2015). The model containing all the variables was first created, including both random and fixed effects. The fixed effects were the sampling number (first to fifth), the type of plant (non-cultivated, open-field cultivated or cultivated under tunnel), and the plant zone where the leaves were collected (bottom, middle, upper), whereas the random effects were the sampling site and the individual plant where leaves were collected. Another similar model was created, with inclusion of the interaction effect between two of the fixed effects: type of plants and plant zone. The two models were compared using the function *anova* with Chi-square (χ^2^) as the test statistic and the second model was significantly better (χ^2^, *p* < 0.05) and hence chosen. The fixed effects of the chosen model were selected by using backward elimination method with the *drop1* function and Chi-square (χ^2^) as the test statistic. Sampling number was then found to not significantly affect the number of phytophagous mites (χ^2^, *p* > 0.05), and hence, a simpler third model was created by excluding this variable. This model was again subjected to backward elimination process and was found to be significantly better than the null model (χ^2^, *p* < 0.05). Therefore, the model without the ‘sampling number’ variable was selected and further optimized using ‘bound optimization by quadratic approximation (BOBYQA)’ optimizer, which was selected through *allFit( )* function. The resulting model was then used for the analysis of intra-plant distribution of phytophagous mites. For analysis of the intra-plant distribution of predatory mites, a similar process was used for constructing a model, with the same fixed and random effect. However, in the backward elimination, numbers of predatory mites were not significantly affected by any of the fixed effects (χ^2^, *p* > 0.05). Therefore, it was not further analyzed.

## Results

### The species of mites

A total of 36 mite samples were successfully sequenced for molecular identification (Table 4, Table S1). Of these, all of them were pooled mite samples, except for three samples of Anystidae species that contained only one mite per sample. Among them, only seven samples were eriophyid mites, all identified to *P. gracilis*. Most of the successfully sequenced eriophyid mites contained more than 50 individuals. *Phyllocoptes gracilis* was confirmed as the only eriophyid mite species sampled through morphological identification of 105 adult females. In the case of spider mites, 23 samples were successfully sequenced for molecular identification, while 29 pairs of adult spider mites were mounted for morphological identification. Sixteen out of the 23 molecular samples were identified as *T. urticae*, and this was confirmed with morphological identification. The remaining molecular samples initially had the highest resemblance (however, only 88% identity, accession number: MN714148) to *Panonychus ulmi* Koch (Acari: Tetranychidae), but morphological identification corrected it to *Neotetranychus rubi* Trägårdh (Acari: Tetranychidae), a species not included in GenBank at the data analysis date (8^th^ August 2023). Therefore, the sequence obtained in this study was submitted to the GenBank on 29^th^ November 2023 as the first molecular sequence for *N. rubi* (Accession number: OR878660). For the predatory mites, *Typhlodromus* (*Typhlodromus*) *pyri* Scheuten (Acari: Phytoseiidae) was identified both molecularly and morphologically. Another species of predatory mite was molecularly identified as Anystidae species (100% identity, accession number: MN352359), but morphologically determined to be *Anystis baccarum* (Linnaeus). Since there was still no record of *A. baccarum* in the GenBank on 29^th^ November 2023, the sequence obtained in this study was submitted to the GenBank on 8^th^ January 2024 as a refinement of molecular identification for *A. baccarum* (Accession number: PP087988). Mites from the family Tydeidae were also identified through molecular identification, but only to family level. All the tydeids sampled were juveniles, and therefore not possible to identify morphologically.

**Table 4:** Molecular identification of mites found on raspberry leaves using comparison of cytochrome c oxidase subunit I gene sequences

	Amplicon size (bp)	Identity (%)	Query cover (%)	Accession (GenBank)
*Phyllocoptes gracilis* (Nalepa)	567–633	98.93–98.95	90–98	QQ869699
*Tetranychus urticae* Koch	606–646	97.52–100.00	100	MG320025
*Neotetranychus rubi* Trägårdh	651–658	100.00	100	OR878660^†^
*Typhlodromus* (*Typhlodromus*) *pyri* Scheuten	651	96.01–97.24	100	JF279173
*Anystis baccarum* (Linnaeus)	598–618	100.00	100	PP087988^†^
Tydeidae	661	94.83	99	MN361611

^†^These are the accession number of the sequences submitted to NCBI GenBank from this study

### Density of mites on raspberry

**Fig. 2:**
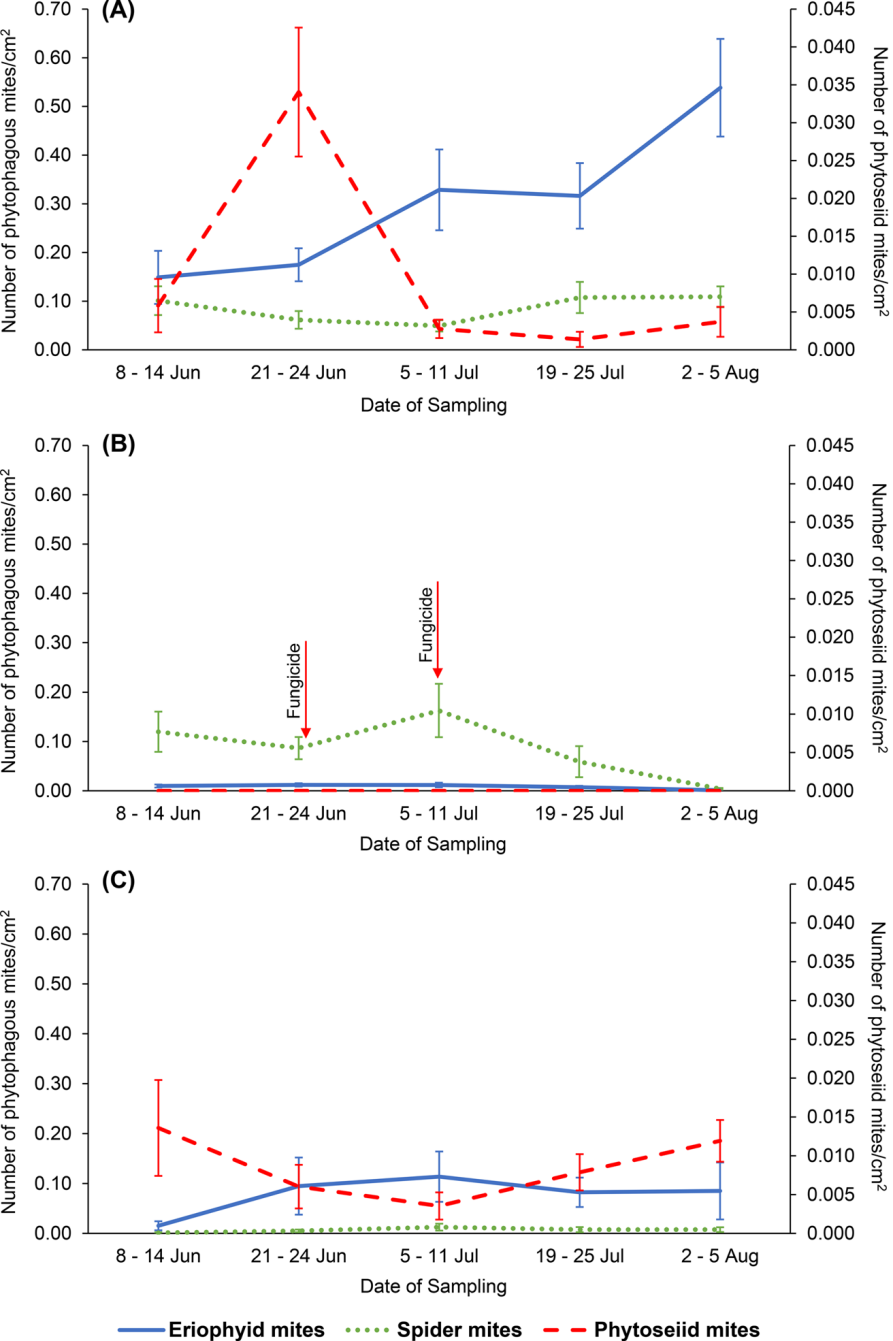
The densities of three mite groups from June to early August 2022, on three types of raspberry: (**A**) non-cultivated raspberry (4 sites), (**B**) open-field cultivated (1 site), and (**C**) cultivated under plastic tunnel (1 site)

In the sampling period, June to August, the floricanes were in flowering and fruiting stage. The temperature was similar during the sampling period at all the sites, which fluctuated around a mean temperature of 16 °C (The Norwegian Meteorological Institute 2023). The densities of phytophagous and phytoseiid mites were generally higher on non-cultivated raspberry than on cultivated raspberry (Fig. 2). The density of eriophyid mites on non-cultivated raspberry was lowest at the beginning (0.149 ± 0.055 mites/cm^2^) and highest at the last sampling, 0.538 ± 0.100 mites/cm^2^ (Fig. 2A). The density of spider mites declined from the beginning (0.101 ± 0.030 mites/cm^2^) to the lowest at third sampling (0.049 ± 0.012 mites/cm^2^) before increasing again to the highest number at the last sampling (0.109 ± 0.021 mites/cm^2^). On the other hand, the phytoseiid mite density increased in the beginning and peaked at the second sampling (0.034 ± 0.009 mites/cm^2^), then declined to the lowest at the fourth sampling (0.001 ± 0.001 mites/cm^2^). Four *A. baccarum* were collected on non-cultivated raspberry, three in the third sampling and one in the last sampling.

In the open-field cultivated raspberry (Fig. 2B), no phytoseiid and *Anystis* mites were found at all. The eriophyid mite density was also very low compared to non-cultivated and tunnel cultivated raspberry. The highest number of eriophyid mites were at the second and third sampling with 0.012 ± 0.003 mites/cm^2^ and 0.012 ± 0.005 mites/cm^2^, respectively, whereas the lowest was at the last sampling, with only 0.001 ± 0.001 mites/cm^2^. In addition to other factors, the application of pesticides, despite not specifically targeting mites, probably still contributed to no predatory mite and low eriophyid mite density in this site. On the other hand, the density of spider mites was the highest in open-field cultivated raspberry, where it peaked at 0.163 ± 0.054 mites/cm^2^ during the third sampling, before declining towards the end of sampling period, with the lowest at the last sampling, 0.004 ± 0.002 mites/cm^2^. The density of spider mites was at the highest even though the application of chlorothalonil (fungicide, 7^th^ July) was only 4 days before the third sampling (11^th^ July).

In the tunnel cultivated raspberry (Fig. 2C), the density of spider mites throughout the sampling period was lower than in the open-field cultivated and non-cultivated raspberry, with only 0.013 ± 0.007 mites/cm^2^ during the peak (third sampling), and 0.001 ± 0.001 mites/cm^2^ at its lowest, during the first sampling. The density of eriophyids seemed to be affected by the predatory mites. When the density of phytoseiid mites was high during the first and last sampling, 0.014 ± 0.006 mites/cm^2^ and 0.012 ± 0.003 mites/cm^2^, the density of eriophyid mites was lower, 0.015 ± 0.009 mites/cm^2^ and 0.085 ± 0.057 mites/cm^2^. The third sampling had the highest number of eriophyid mites, 0.114 ± 0.050 mites/cm^2^, which coincided with the lowest number of predatory mites, 0.004 ± 0.002 mites/cm^2^. Three *A. baccarum* were collected on tunnel cultivated raspberry, one during third sampling while two during forth sampling.

### Analysis of intra-plant phytophagous mite distribution

In cultivated raspberry, both groups of phytophagous mites preferred the upper zone of raspberry floricanes, while in non-cultivated raspberry, they preferred the middle zone (Tables 5 and 6).

**Table 5:** The GLMM output on the eriophyid mite and spider mite population density

Parameter	Eriophyid mites		Spider mites	
	Beta	Standard error	Beta	Standard error
Intercept^†^	1.77098	0.41695	-0.26792	0.85214
Open-field^‡^	-3.24588	0.46730	-0.76045	0.48937
Tunnel^‡^	-1.25232	0.47026	0.79218	0.72400
Middle^§^	0.04636	0.04087	0.32577	0.10124
Upper^§^	-0.56459	0.04855	0.03659	0.10812
Open-field: Middle	0.43914	0.29295	-0.37984	0.15256
Tunnel: Middle	-0.01969	0.13831	-0.75321	0.32521
Open-field: Upper	0.73643	0.31355	0.21764	0.15146
Tunnel: Upper	2.59558	0.11119	0.67806	0.26046

^†^ Intercept is the reference level of the model

^‡^ Open-field represent the type of sampled plants cultivated in an open-field, while tunnel represent the type of sampled plants cultivated under plastic tunnel

^§^ Upper and middle represent the plant zones where respective leaves were sampled

‘:’ (colon) represent the interaction effect of two parameters

**Table 6:** Comparison of intra-plant distribution of eriophyids and spider mites in the three plant types sampled. Analysis based on the GLMM output given in Table 5

	Cultivation Type	Population in comparison to the Bottom zone (%)^†^		Summary^‡^
		Upper	Middle	
Eriophyid mites	Non-cultivated	-43.1	4.7	M > B > U
	Open-field	108.8	55.1	U > M > B
	Tunnel	1240.4	-1.9	U > B > M
Spider mites	Non-cultivated	3.7	38.5	M > U > B
	Open-field	24.3	-31.6	U > B > M
	Tunnel	97.0	-52.9	U > B > M

^†^ Minus symbol (-) represents lower in comparison to the reference, bottom zone

^‡^ U, M, B, represent size of mite population in upper, middle, and bottom zone, respectively, and the ‘>’ symbol represent ‘greater than’

### Presence of RLBV in sampled plants and mites

**Fig. 3:**
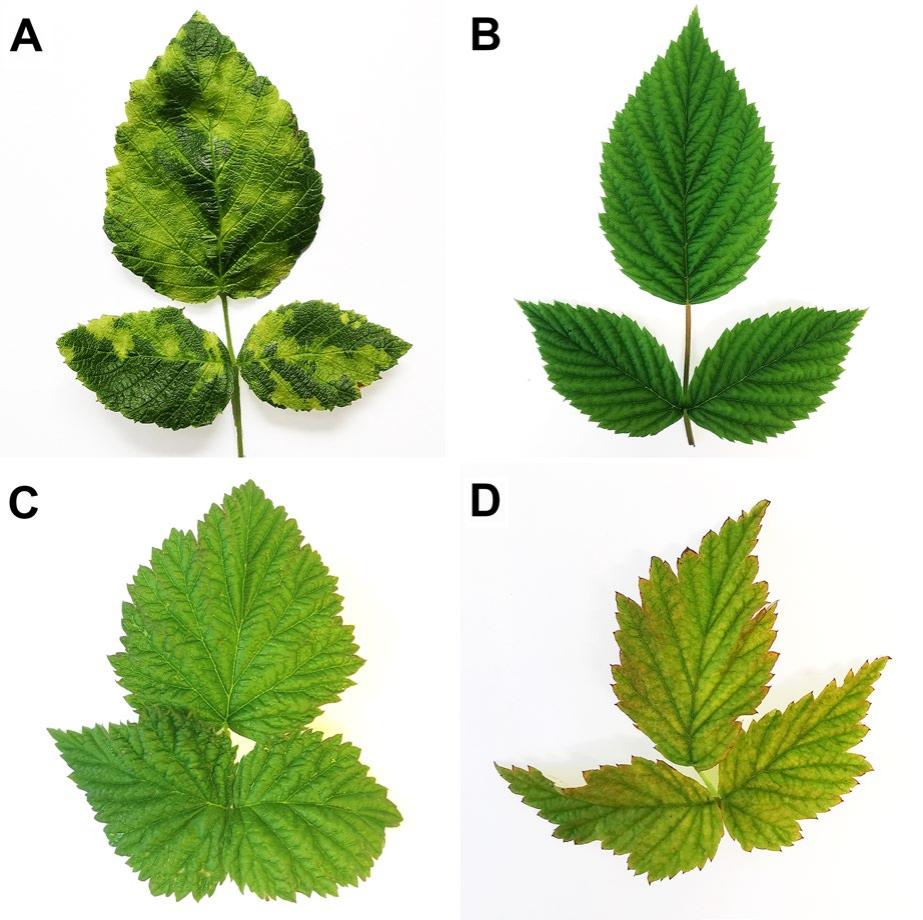
(**A**) Wild raspberry leaf showing raspberry leaf blotch disorder and tested positive for RLBV from laboratory collection in Biology Centre CAS, Czech Republic; (**B**) Cultivated ‘Glen Ample’ raspberry leaf with no symptoms from site 4 that tested negative for RLBV; (**C**) Non-cultivated raspberry leaf with curling and slight interveinal yellowing from site 1 that tested positive for RLBV; (**D**) Non-cultivated raspberry leaf with symptoms of yellowing, slight necrosis and malformation from site 3 that tested positive for RLBV

Out of 453 sampled leaves, eriophyid and spider mites were found on 273 (60.3%) and 196 (43.3%) of these leaves (Table 7), respectively. Figure 3A shows an example of typical raspberry leaf blotch disorder, but none of the sampled leaves exhibited such symptoms and most of them had no obvious virus-like symptoms (Fig. 3B). Out of the 151 sampled plants, only two (1.32%) tested positive for RLBV. Both were non-cultivated raspberry: one plant from site 1, which exhibited curling and slight interveinal leaf yellowing (Fig. 3C), and another from site 3, which had symptoms of yellowing, slight necrosis, and malformation (Fig. 3D). Eriophyid mites were present on both RLBV-positive plants.

Among the 47 groups of tested eriophyid mites, only two (4.26%), both from site 1, were found to be positive for RLBV. Of the 45 groups of spider mites tested for RLBV, four groups from non-cultivated raspberry (one each from site 1 and 2, and two from site 3) and one from open field cultivated raspberry in site 3, were found positive for RLBV. On 10^th^ April 2023, three additional samples of overwintered eriophyid mites from buds on plants in site 1 (more than 30 mites in each sample), were collected. One of these samples tested positive for RLBV with RT-PCR.

The identity of RLBV in four of the RLBV positive mites and plants were confirmed by Sanger sequencing.

**Table 7:** The occurrence of phytophagous mites on sampled raspberry leaves

	Present/total leaves (in %)		Present/total leaves (in %)
	Non-cultivated	Cultivated ‘Glen Ample’	
Eriophyid mites	152/183 (83.1)	121/270 (44.8)	273/453 (60.3)
Spider mites	104/183 (56.8)	92/270 (34.1)	196/453 (43.3)

## Discussion

### Species and density of mites

Even though at least 18 species of phytophagous mites were known to infest raspberry (Tan et al. 2022), only 3 out of those were found in this study, namely *P. gracilis*, *T. urticae* and *N. rubi*. The eriophyid *P. gracilis* is known to be widespread in Europe, North America, and China (Alford 2014; GBIF Secretariat 2022). The remaining two species were spider mites from the family Tetranychidae. Among them, *T. urticae*, is well-known as a highly polyphagous cosmopolitan pest, infesting many agricultural crops worldwide (Assouguem et al. 2022). In contrast, information of *N. rubi* is limited, even though it was reported on *Rubus* spp. over a century ago in Sweden (Trägardh 1915). To date, it has been reported from 15 countries in Europe (Koloniuk et al. 2023; Migeon and Dorkeld 2023), but this study is the first documentation of *N. rubi* in Norway. The co-infestation of *T. urticae* and *N. rubi* in raspberry has also been reported in Poland (Gajek 2003). Therefore, it may be essential to implement measures that target both species in raspberry spider mite management.

Besides the phytophagous mites, two species of generalist predatory mites, *T.* (*T.*) *pyri* and *A. baccarum*, were found. *Anystis baccarum* is a cosmopolitan species, which is widely distributed across the world due to its ability to survive in a wide range of climate conditions. It is found in many geographical locations, and on a wide range of crops (Cuthbertson and Murchie 2010; Cuthbertson et al. 2014; Vincent and Lasnier 2020). However, this may be the first report on its presence in raspberry. *Anystis baccarum* has a wide range of prey (Cuthbertson et al. 2014; Gerson et al. 2003b; Saito et al. 2023; Vincent and Lasnier 2020), and studies of Cuthbertson et al. (2003) and Saito and Brownbridge (2021) have suggested that conserving *A. baccarum* as a native generalist predators can improve the efficacy of biological control against pests. Therefore, a further in-depth study on its potential in managing pests like thrips, aphids, and phytophagous mites in raspberry, should be carried out. Furthermore, leaf sampling was not the best technique for monitoring of *A. baccarum* density due to its fast-moving nature (Cuthbertson and Murchie 2005), therefore, a more comprehensive study on their abundance in raspberry should be conducted. *Typhlodromus* (*T.*) *pyri* is one of the important biological control agents of phytophagous mites in Europe and North America (Edland and Evans 1998; Praslička et al. 2011; Zacharda and Zemek 2013). It is one of the most abundant predatory mite species distributed in eastern and southern part of Norway and found on a total of 43 different plants, including *R. idaeus* (Edland and Evans 1998).

The measures carried out to control pests and diseases in the two cultivated raspberry sites may have influenced the densities of both phytophagous and predatory mites, especially in the open-field cultivated raspberry where both a pyrethroid (Lambda-cyhalothrin, Karate® 5 CS, Syngenta) and a fungicide (Chlorothalonil, Geoxe, Syngenta) were used. This may be one of the reasons for not finding any predatory mites there, whereas such mites were found on non-cultivated raspberry a few meters away. Synthetic pyrethroids are known to be harmful to *T.* (*T.*) *pyri* and *A. baccarum* (Bonafos et al. 2007; Cuthbertson et al. 2014; Edland and Evans 1998; Laurin and Bostanian 2007). The *P. gracilis* population in the open field was consistently low. This mite is known to be more abundant in sheltered conditions (Gordon and Taylor 1976), and the open field was more exposed than all the other sites. Spider mites seems to be more tolerant to the applied agrochemicals, because their density was at the highest during third sampling, that was only 4 days after the application of chlorothanil (the fungicide applied). Both spider mites and eriophyid mite densities were lower at the end of the sampling period than in the beginning, and this may be due to the mites migrating to the primocanes. The migration towards the end of fruiting period, typically in late summer, is commonly observed because raspberry floricanes die after fruiting in the second year (Roy et al. 1999). However, primocanes were not sampled in this study.

The application of vegetable oil with soap during autumn is known to kill overwintering eriophyid mites and prevent leaf blotches the following year (Trandem et al. 2011). This probably explains the notably lower eriophyid mite density in the tunnel (Site 4, cultivated raspberry) at the beginning of the sampling compared to non-cultivated raspberry. But the density also remained much lower than in non-cultivated raspberry throughout the sampling period, and in addition, the density of spider mite was lowest in this site; both phenomena are most probably due to the release of *N. cucumeris* during spring as well as the natural presence of *T.* (*T.*) *pyri* and *A. baccarum*. The release of *N. cucumeris* has previously been noted to reduce populations of *P. gracilis* on Glen Ample raspberry in Finland (Tuovinen and Lindqvist 2014). Interestingly, *N. cucumeris* was not found at all among the collected mites. This could be explained by sensitivity of *N. cucumeris* to light which make them less likely to be found on plants during the day (Weintraub et al. 2007). Another explanation could be the unidirectional intra-guild predation by *A. baccarum*. It has been observed that even when alternative food sources are provided, predation of *A. baccarum* on *N. cucumeris* remains severe (Saito et al. 2023). However, this does not imply a complete annihilation of *N. cucumeris* populations but its foraging behavior can be affected by presence of other predators or the intra-guild predation, leading to their absence on plants (Weintraub et al. 2007). Even though no *N. cucumeris* were sampled, the density of the phytophagous mite was lower when predatory mite density was higher and vice versa in this site suggesting that the predatory mites can control the phytophagous mites. In the non-cultivated raspberry, a similar observation was only noted for the spider mites and not the eriophyids. This could be due to the availability of alternative food sources there for the predators. Generalist predatory mites, such as, *T.* (*T.*) *pyri* and *N. cucumeris*, are known to feed on other mite species, small insects, pollen, and extrafloral nectar (Fathipour and Maleknia 2016; Vangansbeke et al. 2022). Similarly, *A. baccarum* also has a wide range of prey. Further studies on the effect of alternative food sources on the effectiveness of these predatory mites in raspberry should be carried out. In addition, the compatibility of naturally occurring and released natural enemies should be investigated to ensure optimum effectiveness in terms of pest suppression. As approaching 2030, with the adoption of the EU green deal, effort should be placed to assess the feasibility of conservation biological control in managing the phytophagous mites in raspberry because this will reduce dependence on augmentative release of commercial biocontrol agents and hence, reducing the cost of raspberry production.

### Intra-plant phytophagous mite distributions

It has been nearly four decades since Gordon and Taylor (1976) reported that most of the raspberry leaf and bud mite, *P. gracilis*, were found in the upper zone of raspberry plants, presumably to escape the predatory mite *T. (T.) pyri*, which was mostly present in the middle zone. The same trend was observed in the cultivated raspberry in this study, where both eriophyid and spider mites were most likely to be present at the upper zone. Even though, pesticides were applied in the open-field cultivated raspberry, this would not impact the intra-plant distribution but instead the overall density, because the pesticides were applied as blanket spraying. There were several factors affecting the intra-plant distribution of these phytophagous mites. One of the factors is the distribution of predatory mites. Phytoseiid mite was found to prefer the middle and bottom of the plant, where humidity was often higher in these zones due to higher leaf density (Fatnassi et al. 2015). Besides humidity, the movement of phytoseiid mite could be influenced by temperature and light as well as the presence of other predators. For instance, phytoseiid mites often avoid the upper zone during day due to higher temperature and light intensity (Weintraub et al. 2007). The higher density of predators in middle and bottom zone could also result in anti-predation behavior in phytophagous mites, such as those noted in *T. urticae*, causing them to migrate upwards, moving towards the upper zone of plants (Walzer et al. 2009). Unfortunately, in this study, the probability of presence of predatory mites was not significantly influenced by the plant zone, but this may be due to the low number of predatory mites found, limiting the detection of statistical differences. Although no predatory mite was found in the open-field cultivated raspberry, phytophagous mites still prefer the upper zone and this may be due to other factors, such as type of leaves and nutrient distribution within plants. *Tetranychus urticae* was found to prefer feeding on young-fully-opened leaves in the absence of predators (Godinho et al. 2020; Opit et al. 2003). In general, herbivores, especially small arthropods like phytophagous mites, prefers younger leaves due to the higher nutritional value and softer texture which is easier to feed on therefore requiring less energy investment (Caldwell et al. 2016; Nukenine et al. 2010).

However, a similar preference for the upper plant zone was not observed in non-cultivated raspberry, instead both eriophyid and spider mites were most likely to be found in the middle zone. This could be due the different condition the plants were growing in as compared to the cultivated raspberry. Both the cultivated raspberry was ‘Glen Ample’ cultivar and well-fertilized, whereas, non-cultivated raspberry was of unknown cultivars, unfertilized and in constant competition with other vegetations. Smaller raspberry plants with smaller and lesser leaves were typically observed in all the non-cultivated raspberry. Although not specifically investigated in this study, the difference in plant quality is known to be one of the factors that can affect the fitness of phytophagous mites, contributing to change in spatial distribution (Nachman and Zemek 2002). Besides that, with small upper zone leaves, phytophagous mites may be prone to be physically dislodged by rain (Devi and Challa 2019), causing them to move to the middle zone that is more sheltered as it is denser in the presence of other vegetation. Since both the predatory mites found are generalist predators with wide food ranges, the non-cultivated raspberry sites, comprising various other plants in addition to raspberry, should provide more alternative food sources for the predators than cultivated raspberry. For example, the searching behavior of *A. baccarum* which is described as chance-dependent generalist predators (Gerson et al. 2003a), they move in a rapid random zig-zag pattern where any prey that it can find on the path will be captured and consumed (Saito et al. 2023). Besides phytophagous mites, *A. baccarum* is known to feed on wide range of alternative food, which include aphids, thrips, whiteflies, lepidopteran eggs, mealybug and even phytoseiid mites, just to name a few (Saito et al. 2023). Similarly, *T.* (*T.*) *pyri* is also known to feed on alternative food, such as pollen, fungi, and certain plant sap, even when spider mites are present (Sengonca et al. 2004; Tixier 2018; Zemek 2005). Therefore, when more alternative food is available, the phytophagous mites have a higher chance of survival. Besides all the above mentioned factors, there may be other biotic and abiotic factors influencing the intra-plant distribution, and more in-depth studies focusing on the relationship between the factors and the organisms must be carried out to better understand this interaction.

### Phytophagous mites, raspberry leaf blotch disorder, and RLBV

Raspberry leaf blotch disorder was reported to be caused by infestation of *P. gracilis* before the discovery of RLBV (Gordon and Taylor 1976; McGavin et al. 2012). However, it remained uncertain whether the leaf blotch symptoms can be caused both by feeding of *P. gracilis* and infection of RLBV or only by one of them. Our findings from this study may help clarify this uncertainty to a certain extent. Glen Ample, comprising 60% (90/150 plants) of the sampled plants in our study, is a raspberry cultivar known to be very susceptible to raspberry leaf blotch disorder and normally displaying evident blotch symptoms (Bi et al. 2012; Dong et al. 2016; Jevremović et al. 2022; McGavin et al. 2012). In our investigation, *P. gracilis* was found on 44.8% (121 leaves) of the sampled ‘Glen Ample’ leaves (total 270 leaves) and RLBV was not detected using RT-PCR in any of these plants. And, no incident of leaf blotch symptoms was found during the study period, despite June being an optimal period for symptom observation on floricanes based on our previous experience. This result strongly suggests that infestation of *P. gracilis* does not necessarily cause raspberry leaf blotch disorder on raspberry cv. Glen Ample.

RLBV was detected in only two raspberry plants and both were non-cultivated; none of them with typical blotch symptoms. RLBV-positive non-cultivated raspberry with no symptoms or symptoms other than leaf blotch has also been reported from Finland, where leaf yellowing was more common than leaf blotch symptoms (Dong et al. 2016). Two pooled eriophyid mite *P. gracilis* samples and one additional overwintered one, all from site 1, were also found positive for RLBV. This finding aligns with previous research (Dong et al. 2016; McGavin et al. 2012) suggesting that *P. gracilis* is the vector for RLBV. However, it is important to note that *P. gracilis* may not necessarily carry RLBV as 45 out of 47 (95.7%) pooled samples of eriophyids were tested negative for RLBV. The very low incidence of positive findings for RLBV may partly be attributed to the sampling method employed in this study, which differs from the selective sampling of symptomatic plants commonly used when virus occurrence is investigated (Bi et al. 2012; Dong et al. 2016; McGavin et al. 2012).

Five out of 45 samples of pooled spider mites (11%) were found to be positive for RLBV in our study. Although acquisition of virus does not always end up with a transmission, the potential of both the spider mite species as vectors of RLBV should not be overlooked and needs further investigation.

## Conclusions

One species of eriophyid mite, *P. gracilis* and two species of spider mites, *T. urticae* and *N. rubi*, were found on cultivated and non-cultivated raspberry in Viken county, Norway. This is the first report of *N. rubi* in raspberry in Norway. Two species of generalist predatory mites, *A. baccarum* and *T.* (*T.*) *pyri*, occurred. The general trend in the density of the predatory and phytophagous mites indicated that these predators can suppress the phytophagous mites. The potential of *A. baccarum* in conservation biological control in raspberry should be further studied. The phytophagous mites on cultivated raspberry floricanes were observed to prefer the upper zone, whereas on non-cultivated raspberry floricanes, they showed a preference for the middle zone. The presence of *P. gracilis*, without infection of RLBV, may not necessarily lead to the development of raspberry leaf blotch disorder in ‘Glen Ample’ raspberry. Further investigations into the association between raspberry leaf blotch disorder, *P. gracilis* and RLBV are necessary.

### Electronic supplementary material

Below is the link to the electronic supplementary material.

Supplementary Material 1

## Data Availability

The data presented in this study are available on request from the corresponding author.
