# Supplementary material for: The species, density, and intra-plant distribution of mites on red raspberry (Rubus idaeus L.)
Source: Exp Appl Acarol. 2024 Jun 27;93(2):317–37. doi: 10.1007/s10493-024-00930-7 (PMC11269358; doi:10.1007/s10493-024-00930-7)
Supplement: Supplementary file 1 — Supplementary Material 1 [file 10493_2024_930_MOESM1_ESM.docx]

**Table S1** Molecular identification of mites found on raspberry leaves using comparison of cytochrome c oxidase subunit I gene sequences

|  | Lab code | Amplicons (bp) | Identity (%) | Bp identical/  bp compared | Query cover (%) | Accession (GenBank) |
| --- | --- | --- | --- | --- | --- | --- |
|  | | | | | | |
| *Phyllocoptes gracilis* (Nalepa) | P122, P124, P127 | 633 | 98.95 | 567/573 | 90 | QQ869699 |
|  | P123 | 616 |  |  | 93 |  |
|  | P137 | 618 |  |  | 92 |  |
|  | P144 | 626 |  |  | 91 |  |
|  | P139 | 567 | 98.93 | 555/561 | 98 |  |
|  | | | | | | |
| *Tetranychus urticae* Koch | P5, P41 | 646 | 100.00 | 646/646 | 100 | MG320025 |
|  | P6, P12 | 646 | 97.83 | 632/646 |  |  |
|  | P7 | 630 | 98.41 | 620/630 |  |  |
|  | P8 | 626 | 99.36 | 622/626 |  |  |
|  | P9, P10, P35, P77, P82 | 646 | 99.38 | 642/646 |  |  |
|  | P11, P67 | 646 | 99.54 | 643/646 |  |  |
|  | P14, P75 | 646 | 97.68 | 631/646 |  |  |
|  | P43 | 606 | 97.52 | 591/606 |  |  |
|  | | | | | | |
| *Neotetranychus rubi* Trägårdh | P1, P32, P59, P93 | 658 | 100.00 | 658/658 | 100 | OR878660^†^ |
|  | P34 | 651 | 100.00 | 651/651 |  |  |
|  | P45 | 656 | 100.00 | 656/656 |  |  |
|  | P121 | 655 | 100.00 | 655/655 |  |  |
|  | | | | | | |
| *Typhlodromus* (*Typhlodromus*) *pyri* Scheuten | P54 | 651 | 96.01 | 625/651 | 100 | JF279173 |
|  | P86 |  | 97.24 | 633/651 |  |  |
|  | | | | | | |
| *Anystis baccarum* (Linnaeus) | P53, P112 | 618 | 100.00 | 618/618 | 100 | PP087988^†^ |
|  | P60 | 598 |  | 598/598 |  |  |
|  | | | | | | |
| Tydeidae | P61 | 661 | 94.83 | 624/658 | 99 | MN361611 |

^†^These are the accession number of the sequences submitted to NCBI GenBank from this study.
